# Research on optimized multi-exposure image fusion method for improving information entropy in high-brightness region: Based on developing grayscale feature weight matrix

**DOI:** 10.1371/journal.pone.0340650

**Published:** 2026-02-18

**Authors:** Dingran Qu, Yandan Lin

**Affiliations:** 1 School of Intelligent Robotics and Advanced Manufacturing Innovation, Fudan University, Shanghai, China; 2 Intelligent Vision and Human Factor Engineering Center, Shanghai, China; Islamia University of Bahawalpur: The Islamia University of Bahawalpur Pakistan, PAKISTAN

## Abstract

This study serves as a preliminary work for image measurement, aiming to support image-based analysis or measurement tasks of high-brightness light environments. Overexposure can lead to significant loss of information in high-brightness areas of images. To address this issue, this study focuses on the core task of enhancing image information entropy (EN) and proposes a novel multi-exposure image fusion (MEF) method tailored to the characteristics of high-brightness regions. First, low-, medium-, and high-exposure urban outdoor artificial light at night (ALAN) images were simultaneously captured. Based on the brightness characteristics of illuminated regions, a grayscale value weight matrix oriented towards increasing pixel value gradient information was developed. With this as the primary factor and saturation and contrast as supplementary references, an optimized MEF weighting strategy was proposed. Finally, multi-scale fusion was achieved through the Laplacian pyramid. The experimental results in six different scenario show that compared with the classical MEF method, this method significantly improves the EN of the fused ALAN region by 86.49% and increases the mutual information (MI) by 13.88%. It provides an important preprocessing solution for image analysis and measurement tasks.

## 1. Introduction

This study serves as a preliminary work for image measurement, aiming to support image-based analysis or measurement tasks of high-brightness light environments.

With the development of image detection technology, its applications are becoming increasingly widespread. It is gradually providing intelligent, convenient and low-cost solutions for various fields [1–4]. Recent advances in attention mechanisms and feature fusion, such as efficient cross-modal alignment for 3D perception [5] and multi-scale spatial–temporal interaction fusion [6], have further enhanced these capabilities. For light environment measurement tasks, using image detection methods to replace traditional optical measuring instrument can greatly improve measurement efficiency and reduce measurement costs. It is the inevitable trend of the development of intelligent measurement [7–11].

However, due to limitations of imaging devices, the dynamic range of a single image is much lower than the actual light environment. When measuring high brightness areas, such as artificial light at night (ALAN) [12, [13], overexposure and information loss often occur, significantly affecting the expression of key information. Therefore, High Dynamic Range (HDR) imaging technology [14] is needed to address this issue. Multi-exposure image fusion (MEF) [15] is an economical and efficient HDR solution. However, existing MEF methods mainly focus on improving the subjective comfort of image display. Recent works on advanced feature representation and optimization, such as hyperrectangle embedding for debiased prediction [16] and pixel-level noise mining [17], have shown promising results in addressing under-representation and bias in complex visual tasks. Similarly, challenges in 3D reconstruction [18] and image restoration [19] highlight the importance of robust optimization strategies. This study aims to address the issue of information loss in ALAN regions, enhance the information content of images, and facilitate subsequent analysis of high-brightness areas using the images. Image information entropy (EN) can measure the richness of information contained in an image. A higher EN indicates a more dispersed distribution of gray levels and a greater amount of information. Many scholars have adopted it to describe the information content of images [20–22]. Therefore, this paper will primarily focus on enhancing the EN of ALAN regions in images and developing corresponding MEF methods.

Currently, MEF methods are classified into three categories [23]: spatial domain methods, transform domain methods, and deep learning methods [24,25]. This paper focuses on the study of multi-scale fusion methods in the transform domain, achieving MEF through pyramid transformation for multi-scale pixel-level weighted fusion of images. Burt [26] proposed a gradient pyramid model based on directional filtering, which is one of the earliest MEF studies. Mertens T et al. [15] weighted multi-exposure images based on contrast, brightness (pixel value), and saturation of image pixels, and fused them using Laplacian pyramids, forming the classical MEF approach. This method better restores the brightness of the image but performs poorly in severely overexposed areas. Based on this idea, many related optimized MEF methods have been developed.

Li et al. [27] decomposed the image into base and detail layers and calculated weight maps using saliency measures. They refined the weight maps using guided filters, preserving image texture information but suffering from artifacts. Nejati et al. [28] proposed a fast MEF method, using guided filters to decompose the input image into base and detail layers. The brightness component of the input image was then used to combine the base and detail layers based on the blending weights of the exposure function. Singh et al. [29] introduced a novel detail-enhanced exposure fusion method using Nonlinear Transfer Function (NTF) filters to preserve details in very dark and very bright regions. Shen et al. [30] improved the Laplacian pyramid based on enhanced details and structural signals, enhancing the detail expression of color and texture. LZG et al. [31] employed weighted guided image filtering to smooth the Gaussian pyramid of weight maps for all Low Dynamic Range (LDR) images, thereby merging LDR images captured at different exposures. Additionally, they designed a detail extraction component, allowing users to handle fine details in the enhanced images according to their preferences. Kou et al. [32] introduced an edge-preserving smooth pyramid to smooth weight maps, effectively preserving details in the brightest/darkest regions. Following [32], Yang et al. [33] generated virtual images with medium exposure based on input images and used the method from [32] to fuse virtual images and obtain fusion results. Yan et al. [34] utilized a linear fusion framework of Laplacian pyramids to integrate input images captured under different exposure conditions into a fused image, highlighting pixel exposure, contrast, and saturation. Wang et al. [35] designed a simpler multi-scale exposure fusion algorithm in the YUV color space, which can preserve details in the brightest and darkest regions of High Dynamic Range (HDR) scenes. Based on edge-preserving smooth multi-scale exposure fusion algorithm, it can avoid color distortion in the fused image. The resulting enhanced images exhibit significantly enhanced fine details and higher MEF-SSIM values. Qu et al. [36] improved the Laplacian pyramid fusion framework to achieve detail-enhanced fused images. To determine suitable fusion weights, Lin [37] proposed an adaptive search strategy from coarse to fine to search for the optimal weights for multi-scale fusion. Ulucan et al. [38] proposed a simple and effective static image exposure fusion method using weight map extraction based on linear embedding and watershed masking. Xu et al. [39] introduced a new fusion strategy utilizing tensor product and t-svd. The luminance and chrominance channels are fused separately to maintain color consistency, and finally, the fused chrominance and luminance channels are combined to obtain the fused image. Xu et al. [40] presented a fast and efficient image fusion method based on improved weighting functions. Fusion weight maps are calculated by assessing exposure adjustment and relative luminance. By combining pyramid multi-scale decomposition, images of different resolutions are fused to generate the desired HDR image.

Based on the aforementioned research, it is evident that the performance of MEF methods depends on the weight fusion strategy and multi-scale decomposition method. Improving these two aspects can enhance the effectiveness of the fused images and make them more suitable for specific engineering tasks. Pyramid transformation is a commonly used multi-scale decomposition method. Due to different scales and resolutions, the corresponding decomposition layers possess different image characteristic information. Therefore, taking the regional features of image ALAN as an example, this study has designed an optimized exposure fusion weight model. Based on Laplacian pyramid fusion, optimized MEF method for high brightness region features (HRF-OMEF) is realized.

To implement HRF-OMEF, in addition to capturing medium exposure images, it is necessary to capture static images with two additional exposure levels: low and high, while keeping their positions unchanged. When defining the weight calculation model, this study improved the fusion strategy based on the characteristics of the high brightness ALAN area in the images. Using the optimized weight fusion strategy in conjunction with the Laplacian pyramid fusion concept, HRF-OMEF was achieved. Multiple exposure nighttime images around the Bund area in Shanghai were collected for various scenes. After applying HRF-OMEF to these images, multidimensional comparisons including information entropy were conducted against original medium exposure images, low exposure images, classical MEF fusion methods [15], and optimized MEF methods [40], confirming the advanced nature and applicability of the proposed method in this study.

The main contributions of this paper are summarized as follows:

1. A novel MEF method (HRF-OMEF) tailored for high-brightness regions, explicitly targeting EN enhancement for measurement tasks.
2. A data-driven optimization strategy for the brightness weight matrix, designed to maximize gradient information in the fused image.
3. Experimental validation on ALAN scenes showing significant gains in EN over existing methods.

The remaining sections of this paper are arranged as follows: Section 2 introduces the concept of HRF-OMEF method, including ALAN area extraction, optimized weight calculation strategy, and Laplacian pyramid fusion. Section 3 presents the experimental part of this study, including the experimental process, formulation of metrics, comparative experimental results, and analysis of experimental data. The conclusion and future directions of this study will be provided in Section 4.

## 2. HRF-OMEF method

**Fig 1:**
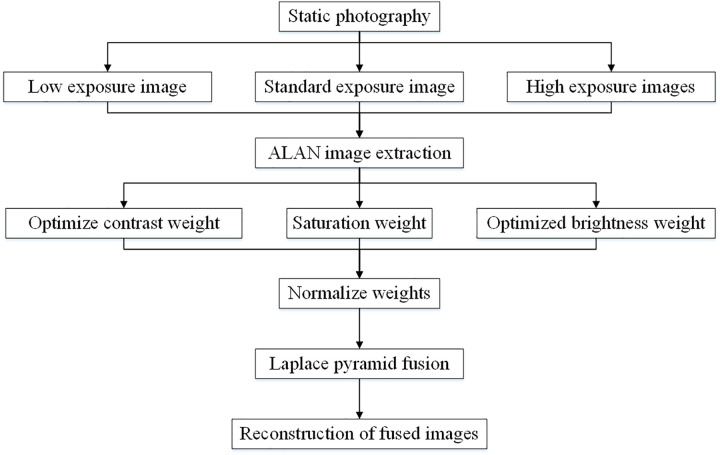
HRF-OMEF.

This study takes the analysis of high brightness ALAN regions as an example. The HRF-OMEF method aims to find the optimal weights for extracting key ALAN information from multiple images captured at different exposure levels, thereby generating new images best suited for ALAN measurement tasks. The method is designed with a core principle of task-specific, data-driven optimization. The methodological framework is built upon three pillars: (1) Only consider the region-of-interest: focusing the computational and optimization efforts solely on the high-brightness ALAN regions. (2) Data-driven weight optimization: the weighting strategy is directly inferred from the data to maximize the gradient information of the fused region, which is intrinsically linked to EN. (3) Multi-scale fusion: employing a Laplacian pyramid-based fusion structure to ensure that the optimized weights are applied consistently across different spatial scales. The HRF-OMEF method is illustrated in Fig 1.

### 2.1. ALAN region extraction

Before fusion, this study roughly extracted the ALAN area of the images. This step serves two purposes: 1. Formulating an optimized weight strategy based on the parameter distribution of the approximate ALAN area; 2. Reducing computational complexity.

The steps are as follows

Firstly, the images are subjected to Gaussian filtering to reduce noise. In order to preserve more realistic color and brightness information, the images are converted to the HSV color mode [41]. In nighttime images, the ALAN area tends to have higher brightness and saturation due to the presence of colored light sources. In this study, pixels with a brightness value (V) greater than a threshold and pixels with a saturation value (S) higher than a threshold in the HSV image are extracted. The remaining pixel values are set to 0 to obtain an approximate ALAN area. In this study, a brightness threshold of 200 and a saturation threshold of 220 (both in the range of 0–255) are set. These thresholds were adjusted based on experiments with multiple datasets. Finally, pixels with a brightness value (V) lower than 100 are filtered out. The ALAN region is extracted from the corresponding positions of the low exposure and high exposure images. In the subsequent optimization weight strategy, specific analysis will be made for this area.

After calculation, the number of non-zero pixels of the six experimental images extracted by ALAN region is reduced by an average of 97.63% compared with the original image. Since the number of pixels to be calculated is greatly reduced, the computational complexity of the fusion method is greatly reduced.

### 2.2. Optimization weight calculation strategy

This study establishes a fusion weight strategy based on three dimensions of image pixels: brightness, saturation, and contrast. Innovative optimization is primarily focused on the brightness weight component.

#### 2.2.1. Calculation of image brightness weight matrix.

The traditional MEF methods typically use predefined, fixed function forms (such as Gaussian function) to calculate the weight. Its core idea is to assign higher weights to pixels with medium exposure levels. However, this strategy fails to adequately account for the specificity of brightness distributions across different scenes, particularly when processing high-brightness scenes (such as ALAN), making it difficult to retain more image information.

**Fig 2:**
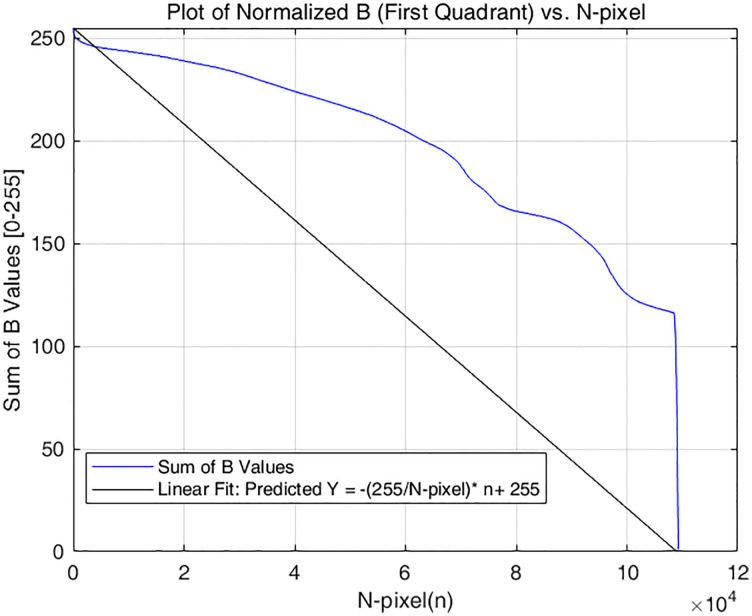
The sum of B values and the target fitting curve.

To address this limitation, this study proposes a data-driven method for calculating the brightness weight matrix to evaluate the relative importance of each pixel based on its brightness value. To establish a data-adaptive brightness weight matrix, this study models it as a parameter optimization problem. In image processing, gradient information is a key indicator for measuring the richness of details. Therefore, a most straightforward optimization objective is to maximize the gradient domain energy of the fused image, thereby enhancing its information entropy [20–22] and increasing the amount of information contained in the image. The derivation process is based on the following steps:

This method utilizes the RGB channels to represent brightness. Taking channel B as an example. Firstly, the B values within the ALAN region from the six exposure images are extracted. Then, they are summed up pixel by pixel and the corresponding coordinates (i, j) are recorded and stored in an array. The summed values are normalized to the range of 0–255 and arranged in descending order to form a coordinate graph. When B = 0, it belongs to an invalid area, so no calculation will be performed. As indicated by the blue line in Fig 2. The horizontal axis represents 1/3 of the sum of B values ${(X_{B}\text{1}}_{n}+{X_{B}\text{2}}_{n}+{X_{B}\text{3}}_{n})/\text{3}$, and the vertical axis represents the pixel index N_pixel, n is the nth pixel from largest to smallest.

As shown in Fig 2, when the pixels are on the baseline $y=-\frac{\text{2}\text{5}\text{5}}{N\_pixel}x+\text{2}\text{5}\text{5}$, the image has denser gradient information. Therefore, when ${X\text{1}}_{n}\times {W\text{1}}_{n}+{X\text{2}}_{n}\times {W\text{1}}_{n}+{X\text{3}}_{n}\times {W\text{1}}_{n}=-\frac{\text{2}\text{5}\text{5}}{N\_pixel}n+\text{2}\text{5}\text{5}$ (the blue line fits the black line), the fused image has the globally optimal brightness gradient information. In order to obtain as much gradient information as possible in the fused image and improve EN. This study uses data-driven methods to obtain the most suitable brightness weight matrix [$W_{\left(i\times j\right)}$], so that the weighted brightness can be closer to the baseline mentioned above.

This study employs a fitting method based on the Mean Squared Error (MSE) loss function to fit the above data and obtain the required weight matrix values, as shown in Equation 1. Here, N represents the number of samples, $Y_{n}$ denotes the true target value of the nth sample, and $\hat{Y_{n}}$ is the predicted target value of the nth sample by the model, as illustrated in Equations 2 and 3. Due to the high brightness characteristics of the ALAN pixels in this study, it is necessary to assign lower weights to the brightness values in most cases during the fitting process. In order to achieve a more uniform weight model, the values of W are constrained within the range of [0, 0.33].


$$MSE=\frac{\text{1}}{N}\sum_{n=\text{1}}^{N}{\left(Y_{n}-\hat{Y_{n}}\right)}^{\text{2}}$$ (1)

$$Y_{n}={X\text{1}}_{n}\times {W\text{1}}_{n}+{X\text{2}}_{n}\times {W\text{1}}_{n}+{X\text{3}}_{n}\times {W\text{1}}_{n}$$ (2)

$$\hat{Y_{n}}=-\frac{\text{2}\text{5}\text{5}}{N\_pixel}n+\text{2}\text{5}\text{5}$$ (3)

The optimization method employs Sequential Least Squares Programming (SLSQP). Iterations cease when the gradient change falls below $\text{1}\times {\text{1}\text{0}}^{-\text{8}}$, thereby obtaining the most suitable weight values. The pixel coordinates (i, j) corresponding to each weight are recorded at the corresponding position in the matrix. Finally, the R, G, and B weight matrices are multiplied to obtain the final weight matrix. Therefore, the brightness weight of each pixel is represented as shown in Equation 4, where ${{Wk}^{B}}_{i,j}$ denotes the value of the B channel weight matrix W at position (i, j) for the k-th input image.


$$E_{i,j,k}={{Wk}^{B}}_{i,j}\times {{Wk}^{G}}_{i,j}\times {{Wk}^{R}}_{i,j}$$ (4)

#### 2.2.2. Calculation of saturation weight.

Regions with higher saturation have a lower degree of overexposure or underexposure, and a higher relative quality. Therefore, we give greater weight to regions with higher saturation. On the contrary, a lower saturation value indicates more severe overexposure or underexposure, thus resulting in a smaller weight proportion. Saturation weight can be represented by the standard deviation of the RGB channels, as shown in Equations 5. $S_{k,i,j}$ represents the saturation weight index, where $I_{k,i,j}^{R}$, $I_{k,i,j}^{G}$, and $I_{k,i,j}^{B}$ denote the R, G, and B channel values at position (i,j) in the k-th image, respectively. The calculation of $M_{k,i,j}$ is shown in Equations 6.


$$S_{k,i,j}=\sqrt{\frac{{\left(I_{k,i,j}^{R}-M_{k,i,j}\right)}^{\text{2}}+{\left(I_{k,i,j}^{G}-M_{k,i,j}\right)}^{\text{2}}+{\left(I_{k,i,j}^{B}-M_{k,i,j}\right)}^{\text{2}}}{\text{3}}}$$ (5)

$$M_{k,i,j}=\frac{I_{k,i,j}^{R}+I_{k,i,j}^{G}+I_{k,i,j}^{B}}{\text{3}}$$ (6)

#### 2.2.3. Calculation of contrast weight.

This method also preserves regions with higher contrast. The contrast weight is determined based on the gradient magnitude extracted by the Laplace operator, as shown in Equation 7. $C_{k,i,j}$ represents the contrast weight index, where $I_{k,i,j}$ denotes the pixel value at position (i,j) in the k-th image. A higher contrast indicates more details in the pixel region, resulting in a larger weight.


$$C_{k,i,j}=\left|I_{k,i-\text{1},j}+I_{k,i+\text{1},j}+I_{k,i,j-\text{1}}+I_{k,i,j+\text{1}}-{\text{4}I}_{k,i,j}\right|$$ (7)

#### 2.2.4. Normalization of weights.

After obtaining the weights of the above three dimensions, the final weight corresponding to each pixel is:


$$W_{i,j,k}=C_{i,j,k}\times S_{i,j,k}\times E_{i,j,k}$$ (8)

In order to ensure that the sum of weights for multiple images in the same position is 1, it is necessary to normalize the weights in the dimension of image quantity:


$${\hat{W}}_{ij,k}=\frac{W_{ij,k}}{\sum_{k^{\prime }=\text{1}}^{N}W_{ij,k^{\prime }}}$$ (9)

### 2.3. Multi-scale fusion based on laplacian pyramid

Due to the varying exposure times of each image, the absolute intensity of pixels differs, direct fusion will lead to too large gray jump in the area with sharp weight transition and noise. Therefore, this study employed a fusion method based on Laplacian pyramid [15], which involves multi-scale decomposition fusion of the images.

#### 2.3.1. Gaussian pyramid decomposition.

First, the input image sequence is convolved with a Gaussian filter to create a Gaussian pyramid for each input image. The decomposition formula is given by Equation 10.


$$Text.\;G_{l}(x,y)=\sum {\mathrm{\backslash nolimits}}_{i=-\text{2}}^{\text{2}}\sum {\mathrm{\backslash nolimits}}_{j=-\text{2}}^{\text{2}}w(i,j)G_{l-\text{1}}(\text{2}x+i,\text{2}y+j)\;$$ (10)

$$\left(\;\text{0}\leq l\leq L_{ev}-\text{1},\;\text{0}\leq x\leq C_{l}-\text{1},\;\text{0}\leq y\leq R_{l}-\text{1}\right)$$

Where, $G_{l}$ is the image of layer l of the Gaussian pyramid. w(i,j) is the value of line i and column i of the Gaussian filter template. $L_{ev}$ is the number of layers of the Gaussian pyramid. In this study, $L_{ev}$ =9. $C_{l}$, $R_{l}$ is the total number of rows and columns of the layer l image.

#### 2.3.2. Laplacian pyramid decomposition.

In order to solve the problem of loss of image information during down-sampling Gaussian pyramid, Laplacian pyramid decomposition is applied to multi exposure image sequence [42].

The layer l Laplacian pyramid $L_{l}$ is equal to the layer l image of the Gaussian pyramid $G_{l}$ minus $expand({\hat{G}}_{l}(x,y))$. The calculation is as shown in Equation 11. $expand({\hat{G}}_{l}(x,y))$ is the up-sampling of the layer l of the Gaussian pyramid. As shown in Equation 12, 13. Z is integer type.


$$L_{l}=\left\{ \;\;\;\begin{array}{c} G_{l}-expand\left({\hat{G}}_{l}(x,y)\right),\;\text{0}\leq l\leq L_{ev}-\text{1}\\ G_{L_{ev}},l=L_{ev} \end{array}\right.\;$$ (11)

$$\begin{array}{c} expand({\hat{G}}_{l}(x,y))=\text{4}\sum {\mathrm{\backslash nolimits}}_{i=-\text{2}}^{\text{2}}\sum {\mathrm{\backslash nolimits}}_{j=-\text{2}}^{\text{2}}{\hat{G}}_{l}\left(\frac{\left(x+i\right)}{\text{2}},\frac{\left(y+j\right)}{\text{2}}\right)w(i,j) \end{array}$$ (12)

$${\hat{G}}_{l}\left(\frac{\left(x+i\right)}{\text{2}},\frac{\left(y+j\right)}{\text{2}}\right)=\left\{ \;\;\;\;\begin{array}{c} {\hat{G}}_{l}\left(\frac{\left(x+i\right)}{\text{2}},\frac{\left(y+j\right)}{\text{2}}\right),\;\frac{\left(x+i\right)}{\text{2}},\frac{\left(y+j\right)}{\text{2}}\in z\\ \text{0},else \end{array}\right.\;$$ (13)

#### 2.3.3. Image fusion and reconstruction.

After fusing the Gaussian and Laplacian pyramids of the images at corresponding levels, the upper-level images of the fused pyramid are up-sampled. The up-sampled image is then added to the lower-level Laplacian pyramid image, and this process continues for all levels to complete the fusion. As shown in Equation 14. H represents the final fused image, $FI_{l}$ represents the fused image data of layer l, up represents up-sampling, $L_{ev}$ represents the number of pyramid levels, which is 9 in this study.


$$\begin{array}{c} H=\sum {\mathrm{\backslash nolimits}}_{l=L_{ev}-\text{2}}^{\text{0}}FI_{l}+up(FI_{l+\text{1}}) \end{array}$$ (14)

$FI_{l}$ is the fusion image data of layer l, as shown in Equation 15. $W_{k,l}$ is the layer l data of the kth weighted image. $L_{k,l}$ is the layer l data of the Laplacian pyramid of the kth multi exposure image. N is the number of images.


$$\begin{array}{c} FI_{l}=\sum {\mathrm{\backslash nolimits}}_{k=\text{1}}^{N}W_{k,l}L_{k,l},\text{0}\leq l\leq L_{ev}-\text{1} \end{array}$$ (15)

Subtracting the fused Gaussian pyramid from the corresponding level of the fused Laplacian pyramid, and then reconstructing the image through inverse operations in reverse order, results in the HDR image.

## 3. Experimental results and analysis

### 3.1. Objective evaluation indicators

Traditional MEF image quality assessment mainly focuses on image features, structural similarity, and human subjective perception [43]. This study emphasizes the detail information in the ALAN area, hence requiring a more objective quantitative evaluation model. The evaluation indicators of MEF method in static scenes can be referred to in reference [23].

In order to adapt to the task of ALAN image measurement in this study, it is necessary to assess whether the fused image contains more detail information of the target area. Therefore, this study selects 3 commonly used metrics as reference standards: Percentage of overexposed pixels, mutual information (MI) [44], EN of the ALAN portion. The percentage of overexposed pixels refers to the percentage of pixels with a pixel value of 255 in the effective pixels of the ALAN region, a higher percentage of overexposed pixels, indicates poorer image quality after fusion. Higher values of MI, EN in the ALAN area indicate that the fused image contains more target information, thus indicating higher quality.

#### 3.1.1. Mutual information.

The MI reflects the amount of information of the ALAN region in the fused image obtained from the input image sequence. In this study, MI is calculated as shown in Equation 16.


$${{MI}_{F}^{ABC}=I}_{FA}(f;a){+I}_{FB}(f;b){+I}_{FC}(f;c)$$ (16)

Where A, B and C are the three multi exposure images input, and F is the fused image. The MI between F and the input images are calculated as shown in Equation 17–19. Where, $p_{FA}(f,a)$ is the joint distribution of image F and A. $p_{F}(f)$ and $p_{A}(a)$ are the edge distribution of image F and A, respectively. This data can be obtained by normalizing the joint histogram and edge histogram of image F and A.


$$\begin{array}{c} I_{FA}(f;a)=\sum {\mathrm{\backslash nolimits}}_{f,a}p_{FA}(f,a){log}_{\text{2}}\frac{p_{FA}(f,a)}{p_{F}(f)p_{A}(a)} \end{array}$$ (17)

$$\begin{array}{c} I_{FB}(f;b)=\sum {\mathrm{\backslash nolimits}}_{f,b}p_{FB}(f,b){log}_{\text{2}}\frac{p_{FB}(f,b)}{p_{F}(f)p_{B}(b)} \end{array}$$ (18)

$$\begin{array}{c} I_{FC}(f;c)=\sum {\mathrm{\backslash nolimits}}_{f,c}p_{FC}(f,c){log}_{\text{2}}\frac{p_{FC}(f,c)}{p_{F}(f)p_{C}(c)} \end{array}$$ (19)

#### 3.1.2. Image information entropy.

The EN of the ALAN region indicates the information richness of the region in the image, which is the main improvement goal of this study. EN calculation is shown in Equation 20. ${P(k}_{i})$ indicates the probability when the pixel in the image is i, n∈ [0,255].


$$\begin{array}{c} EN=-\sum {\mathrm{\backslash nolimits}}_{i=\text{1}}^{n}{P(k}_{i}){ln}_{\text{2}}{P(k}_{i}) \end{array}$$ (20)

### 3.2. Comparison of experimental results and analysis

Due to the high brightness and high number of overexposed pixels characteristic of ALAN, this experiment included low exposure images in the comparison group. In order to verify the superiority of the HRF-OMEF method in ALAN measurement tasks, the fused images were tested and compared with medium exposure images, low exposure images, classical MEF fusion images [15], and optimized MEF images [40].

#### 3.2.1. Experimental data.

This study employed six sets of urban ALAN scene images for testing, all collected from the vicinity of the Bund area in Shanghai.

The photos were taken during the night from 20:00–21:00, under clear weather conditions. Exposure levels were defined by varying exposure times. The camera used for the experiment was a Canon EOS 70D. In this experiment, exposure times for low, medium and high exposures were set to 1/250s, 1/4s, and 2s, respectively. The ISO was fixed at 100, and the aperture value was fixed at 5.6. To simulate the viewpoint of a ground-level observer, the camera was mounted on a tripod at a height of approximately 1.6m above the ground. Exposure adjustments were made while keeping the camera position fixed to avoid overlapping images.

As this study focuses on the information content of the ALAN area in the fused images, testing was conducted only on the extracted ALAN area.

#### 3.2.2. Result analysis.

Through experimental testing, a comparison of different fusion images was conducted across three dimensions: MI, EN and percentage of overexposed pixels. The results for the six test scenarios are shown in Tables 1–6.

**Table 1:** Comparative experimental results analysis – test scenario 1.

Image type	Percentage of overexposed pixels	MI	EN
Medium exposure	23.16%		0.2605
Low exposure	0%		0.2818
Classic MEF	5.92%	0.3657	0.3080
Optimized MEF	0%	0.3739	0.3026
HRF-OMEF	0%	0.3897	0.4896

**Table 2:** Comparative experimental results analysis – test scenario 2.

Image type	Percentage of overexposed pixels	MI	EN
Medium exposure	3.38%		0.3219
Low exposure	0.25%		0.1913
Classic MEF	1.65%	0.3863	0.3400
Optimized MEF	0%	0.3966	0.3218
HRF-OMEF	0.36%	0.4016	0.6885

**Table 3:** Comparative experimental results analysis – test scenario 3.

Image type	Percentage of overexposed pixels	MI	EN
Medium exposure	7.04%		0.6521
Low exposure	0%		0.6215
Classic MEF	2.66%	0.7149	0.6893
Optimized MEF	0%	0.7665	0.6708
HRF-OMEF	0%	0.7953	1.1028

**Table 4:** Comparative experimental results analysis – test scenario 4.

Image type	Percentage of overexposed pixels	MI	EN
Medium exposure	0.35%		0.2015
Low exposure	0%		0.1641
Classic MEF	0.34%	0.1641	0.2799
Optimized MEF	0%	0.2243	0.3531
HRF-OMEF	0%	0.2540	0.4824

**Table 5:** Comparative experimental results analysis – test scenario 5.

Image type	Percentage of overexposed pixels	MI	EN
Medium exposure	0.14%		0.2381
Low exposure	0%		0.1754
Classic MEF	0.10%	0.2636	0.2728
Optimized MEF	0%	0.3069	0.4372
HRF-OMEF	0%	0.3139	0.5840

**Table 6:** Comparative experimental results analysis – test scenario 6.

Image type	Percentage of overexposed pixels	MI	EN
Medium exposure	6.99%		0.1821
Low exposure	0.10%		0.1444
Classic MEF	1.30%	0.2103	0.2144
Optimized MEF	1.36%	0.2289	0.3127
HRF-OMEF	0.29%	0.2425	0.5772

From Tables 1–6, it can be observed that in the six experimental scenarios, the HRF-OMEF method proposed in this study has a very low proportion of overexposed pixels in the fused images. Compared with Classic MEF [15], the MI value increased by an average of 13.88% and the EN value increased by an average of 86.49%. Compared with the optimized MEF [40], the MI value increased by an average of 4.35% and the EN value increased by an average of 63.64%.

To statistically validate the performance improvement, paired-sample t-tests were conducted on the results from the six experimental scenarios. The analysis revealed that the proposed HRF-OMEF method yielded a statistically significant increase in both Information Entropy (EN) and Mutual Information (MI) compared to the baseline methods. Specifically, as shown in Table 7, when compared to the Classic MEF method, the improvements were highly significant for both EN (p < 0.001) and MI (p < 0.05). Similarly, significant improvements were observed over the Optimized MEF method for EN (p < 0.01) and MI (p < 0.05). All p-values were well below the 0.05 significance threshold, confirming that the superior performance of HRF-OMEF is not due to random chance.

**Table 7:** Results of paired-sample t-tests for EN and MI.

Comparison group	Indicator	t-value	p-value	Significance
HRF-OMEF vs Classic MEF	EN	8.03	0.0005	Significant(p < 0.001)
HRF-OMEF vs Classic MEF	MI	3.89	0.0115	Significant(p < 0.05)
HRF-OMEF vs Optimized MEF	EN	5.05	0.0039	Significant(p < 0.01)
HRF-OMEF vs Optimized MEF	MI	3.86	0.0118	Significant(p < 0.05)

Therefore, compared to the control group, the HRF-OMEF method shows a significant improvement in information entropy. Additionally, it demonstrates favorable performance in both MI and percentage of overexposed pixels. Thus, HRF-OMEF method significantly improves the information entropy of the ALAN region of the generated image, so that it contains more detailed information. It has important practical application value for image high brightness area analysis and measurement tasks.

## 4. Conclusion

Overexposure can result in a substantial loss of valuable information in the high-brightness regions of an image, rendering image analysis difficult. To address this problem through the production of HDR images, this paper proposes a novel multi-exposure image fusion method specifically optimized for the characteristics of high-brightness regions. Taking the ALAN region as an example, the high brightness ALAN region of the image was analyzed, and the weighting strategy for multi-exposure image fusion is specifically improved. The final fusion is achieved based on the Laplacian pyramid multi-scale model. Through experiments, comparisons were made across three dimensions: percentage of overexposed pixels, Mutual Information, and Entropy in the high brightness ALAN region. These comparisons were made against the original images, low-exposure images, fused images using 2 existing MEF methods and HRF-OMEF method. The HRF-OMEF method proposed in this paper demonstrates significant improvement in terms of information entropy, while also showing varying degrees of improvement in the other two indicators. Therefore, this method can retain more image information when processing high brightness image regions such as ALAN. Generating images with more informative pixels is of great significance for image analysis and measurement tasks.

The HRF-OMEF method has good performance in improving EN in high brightness areas of images. However, However, it still has certain limitations and there are areas for improvement in future research:

1. Although an increase in EN indicates a growth in information, this information may include noise. Therefore, more precise metrics will be researched and developed.
2. Deep convolutional networks will be applied to ALAN area extraction in future research. After annotating a large dataset manually, the ALAN area extraction model will be trained to improve speed, accuracy, generalization, and robustness.
3. The process calculation of this method is relatively large. Subsequently, it is necessary to reduce spatial and temporal complexity by adjusting parameters or optimizing algorithms.
4. This study takes ALAN as an example for analysis, and in the future, stronger generalization algorithms will be developed to be applied to more high-brightness scenes.
5. The Laplacian operator may not be able to fully capture complex texture changes in certain scenarios, which reflects the trade-off between computational efficiency and thorough detail extraction. Therefore, researching and integrating more robust computing methods (e.g., Sobel, guided filter-based contrast) to further enhance the detail preservation ability of fusion algorithms, especially in areas with fine textures, is a promising direction for our future research.
